# Re-irradiation in the treatment of patients with cerebral metastases of solid tumors: retrospective analysis

**DOI:** 10.1186/1748-717X-9-4

**Published:** 2014-01-03

**Authors:** Maike Scharp, Henrik Hauswald, Marc Bischof, Juergen Debus, Stephanie E Combs

**Affiliations:** 1 Department of Radiation Oncology, University of Heidelberg, Im Neuenheimer Feld 400, Heidelberg, Germany

**Keywords:** Re-irradiation, Brain metastasis, Cerebral metastasis, Whole brain radiotherapy, Palliative care, Solid tumors, Effectiveness, Safety

## Abstract

**Background:**

Goal of this retrospective analysis was to evaluate the role of repeat whole brain radiotherapy in the palliative care of patients with brain metastases due to solid tumors.

**Methods:**

Data regarding demographic data, primary tumor, metastasis, radiotherapy and symptoms were compiled on 134 patients with cerebral metastases that received repeat whole brain radiotherapy (WBRT) in our clinic between 2002 and 2011.

**Results:**

The analyzed group consisted of 63 (47%) women and 71 (53%) men with a median age of 57 at the start of re-irradiation. Most frequent primary site was the lung (87%).

Sixty patients with lung cancer received the first WBRT prophylactically. At the time of re-WBRT 81% of all patients suffered from additional extracerebral metastases.

Time between first and second WBRT was a median of 13.4 months. Full dose for the first WBRT was 30 Gy in 2.0 Gy single dose, for the second 20 Gy in 2.0 Gy single dose.

At the start of the Re-WBRT 81 patients (60.4%) had mild, 32 (23.9%) severe neurological symptoms, 21 patients (15.7%) were asymptomatic. The median Karnofsky performance status was 70%. Overall, re-WBRT was tolerated satisfactorily. Main side effects were fatigue, erythema and focal alopecia, 10% of patients discontinued treatment before reaching the planned dose. Median survival was 2.8 months since the end of the re-WBRT with good performance status at the start of the re-irradiation being a key indicator for longer survival.

Fifty-two patients (39%) showed a clinical improvement of neurological symptoms after the therapy, 59 patients (44%) remained stable, 23 patients (17%) showed worse symptoms.

**Conclusions:**

From this large patient collective we were able to show that re-WBRT can be an important therapeutic option with low rate of acute side effects for patients in adequate general condition.

## Background

Cerebral metastases pose a significant health care problem. 20-40% of cancer patients develop brain metastases during the course of their disease [1] and numbers are likely to increase due to improved systemic treatment and resulting increase in survival times. Prognosis for these patients is generally poor with a median survival of 4–5 months [2], however, patients often present in overall acceptable performance status, with a strong wish for additional treatment. While treatment options include surgery, chemotherapy and irradiation, whole brain radiotherapy (WBRT) is considered standard of care especially for patients with multiple metastases [3,4]. Unfortunately, in general, the effect is not very enduring, and time to clinical progression after WBRT lies between 6 and 13 weeks [5].

Treatment options in this recurrent situation, especially for patients with multiple lesions, are limited, including re-irradiation, surgery, best supportive care and, to a smaller degree, chemotherapy. Treatment should be decided individually based on the patient’s general performance status, extracranial disease, number of metastases, primary malignancy and previous treatments [6].

While patients with a small number of metastases might be treated by stereotactic radiosurgery [7,8], options are extremely limited in the case of multiple metastases.

Re-WBRT can be an important treatment option, especially for patients with stable extracranial disease and sufficient general performance status. However, most institutions are hesitant to apply a second course of WBRT due to a fear of toxicity, thus reports on Re-WBRT and treated patient numbers are relatively low. Comparative analyses are missing and patient numbers in the existing studies are not sufficient to lead to general recommendations [9].

In our institution, the indication for Re-WBRT is seen depending on overall performance status of the patients, underlying disease, and other comorbidities. In the present analysis we evaluated our group of patients with recurrent or progressive brain metastases treated with Re-WBRT with the objective to examine survival following re-irradiation. The group represents one of the larger groups of patients published to date treated in a single center with a homogenous treatment algorithm.

## Methods

We identified 134 patients in our cancer center database that had received Re-WBRT in our department between 2002 and 2011. Patients treated for malignant myeloma, metastases of the skull or primary brain tumors were excluded. We also excluded all treatments that were not whole brain radiotherapy, e.g., stereotactic radiosurgery, partial-brain radiotherapy etc. The study was approved by the ethics committee Heidelberg.

From the patient charts we retrieved the following data: age, primary malignancy including stage and grading, extracranial disease status, first diagnosis and number of brain metastases, dose and fractionation of first and second WBRT, symptoms before and after irradiation, response to re-irradiation, additional treatment, side effects of irradiation, progression of metastases, treatment as in- or outpatient.

Symptoms were classified as asymptomatic, mild symptoms and severe symptoms based on description in the patient charts. Signs of intracerebral pressure, hemiparesis or general seizures where classified as severe symptoms, neurocognitive testing was not performed on a regular basis.

Karnofky performance status (KPS) was taken from patient records for 50% of patients. If no KPS was indicated in the records, the value was estimated based on the description of the patient’s clinical status at the relevant time point.

All patients were assigned an RPA (recursive partitioning analysis) class based on KPS, local control, extracranial metastases status and age. Patients ≤ 65, with a KPS ≥ 70, local control and no extracranial metastases were assigned RPA class I. Patients with a KPS < 70 were assigned RPA class III, all others were assigned to group II.

Progression after therapy was based on clinical evaluations or imaging where possible. Patients were invited to regular follow-up visits till their death with a median follow-up of 2.1 months.

Date of death was obtained from medical and official records.

Statistical analyses were conducted with SPSS version 16.0.1 (SPSS Inc., Chicago, IL, USA) using Cox regression analyses (stepwise backwards, p-in 0.05, p-out 0.1), log-rank test and Kaplan-Meier method. If not declared otherwise, survival time was defined as the time period between the last day of re-irradiation and date of death. Statistical significance was defined as p < 0.05.

## Results

134 patients received a second course of whole brain radiotherapy in our institution between 2002 and 2011 with the number of re-irradiations/year increasing significantly over the years. Patient characteristics are listed in Table 1. 63 patients (47%) were female and 71 (53%) were male. The median age at the start of the Re-WBRT was 57 (range 31–82). The main primary tumor site was lung (117 patients, 87%) followed by breast (12 patients, 9%) and others (two malignant melanoma, two carcinoma of unknown primary and one lung cancer with synchronous malignant melanoma).

**Table 1:** Patient characteristics

		**n**	**%**
Sex			
	Male	71	53%
	Female	63	47%
Age			
	18-49 years	27	20%
	50-59 years	51	38%
	≥60 years	56	42%
Primary Site			
	Lung *thereof SCLC*	117 *81*	87% *60%*
	Breast	12	9%
	Other	5	4%
Year of re-irradiation			
	2009 or before	55	41%
	2010	41	31%
	2011	38	28%
RPA class			
	RPA 1	6	5%
	RPA 2	83	62%
	RPA 3	45	34%

At the time of the Re-WBRT the primary tumor was locally controlled in 36 cases (27%) and not controlled in 95 cases (71%), for 3 patients no relevant data was available. 43 patients (32%) had additional metastases in one extracerebral location, 66 patients (49%) had metastases in at least two extracerebral locations prior to re-irradiation, only 25 patients (19%) had no additional metastases.

The median dose for the first WBRT was 30 Gy in 2.0 Gy fractionation (range 30–40 Gy). The median time interval between the first and the second WBRT was 13.4 months (range 3.4-58.8 months).

Sixty patients with lung cancer received the first whole brain irradiation prophylactically, the other 74 had radiologically confirmed cerebral metastases, the median time interval between the first and the second WBRT were almost the same for the two groups (13.5 and 13.4 months).

The median dose for the second WBRT was 20 Gy in 2.0 Gy fractions (range 2–30 Gy).

The mean Karnofsky performance status (KPS) at the time of retreatment was 70% (range 40-100%) both for only the group that had a KPS indicated in records and for the whole cohort. The majority of patients (81 patients, 61%) had mild neurological symptoms, 32 patients (24%) had severe symptoms, 21 patients (16%) were asymptomatic.

**Figure 1:**
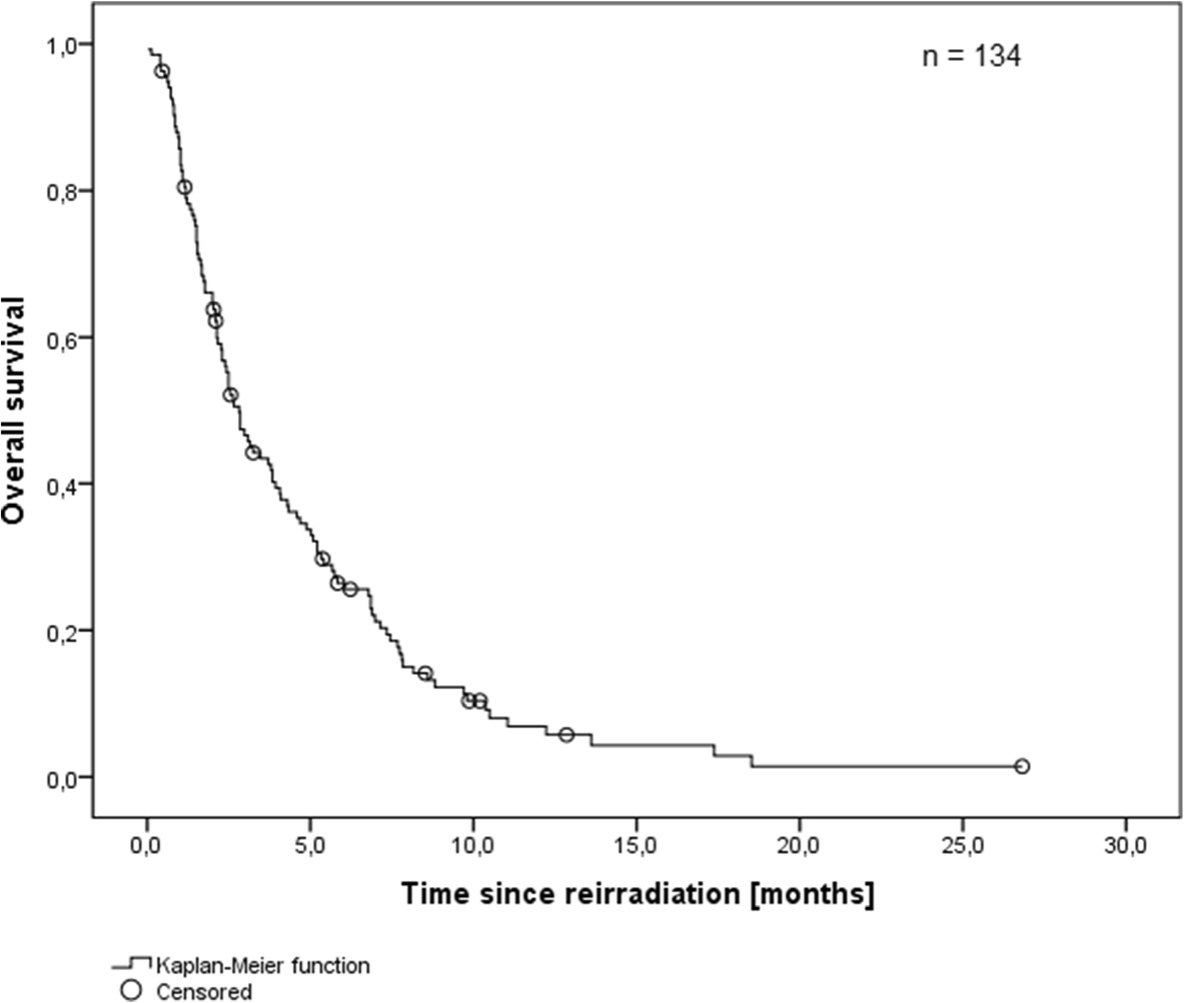
Overall survival after re-irradiation (Kaplan-Meier’s estimation).

Based on KPS, age, local control and extracranial metastasis at the time of retreatment, the majority of patients (83 patients, 61.9%) was classified as RPA class II, 6 patients (4.5%) were classified as RPA class I, 45 patients (33.6%) were classified as RPA class III. Median survival was 2.8 months (0–28 months) since start of re-irradiation (Figure 1), 10.2 months since the initial diagnosis of brain metastases and 23.9 months since primary diagnosis.

At the end of the therapy 52 patients (39%) showed clinical improvement of neurological symptoms, 59 patients (44%) had no relevant change, 23 patients (17%) showed more severe symptoms.

**Figure 2:**
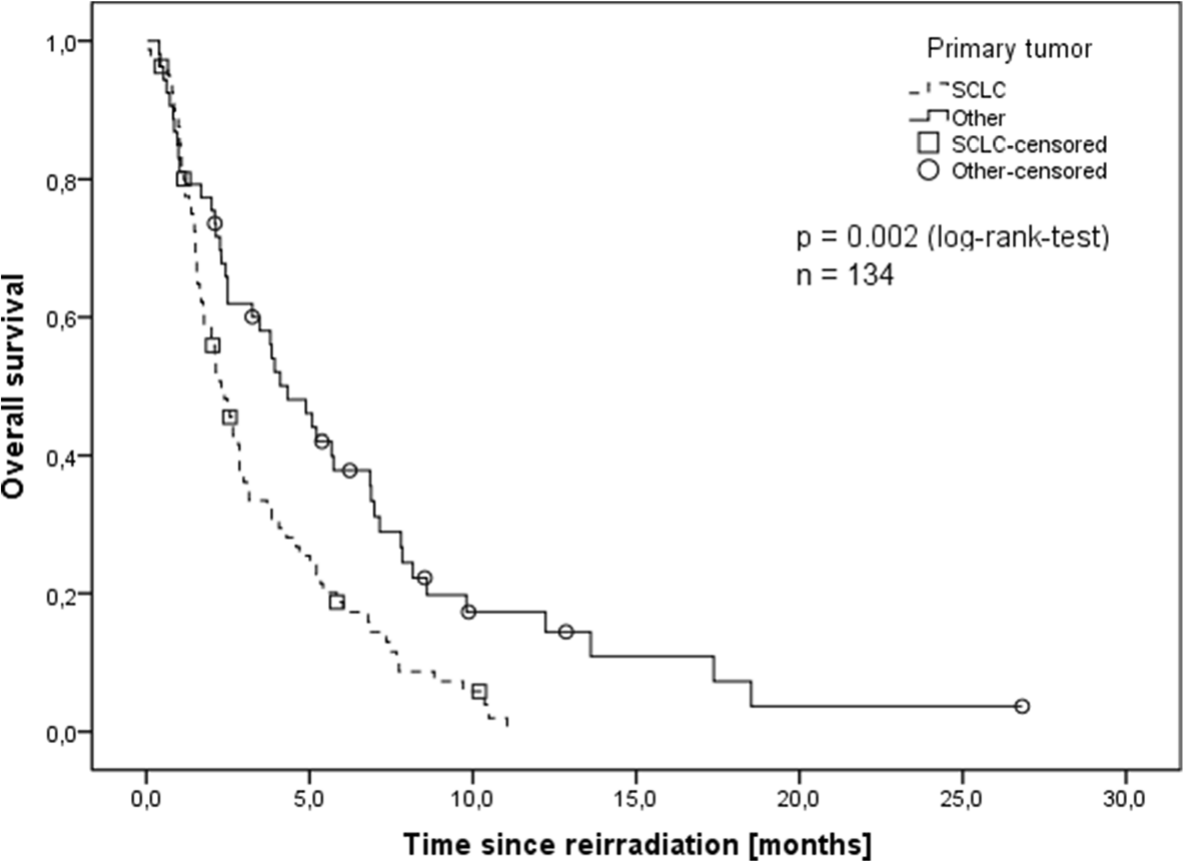
**Overall survival depending on primary tumor site (Kaplan-Meier’s estimation).** SCLC with significantly worse prognosis (p = 0.002, log-rank-test).

Patients with small cell lung cancer (SCLC) had the shortest overall survival (median 2.3 months) with the other primary sites ranging between 3.9 and 4.3 months with SCLC being a significant negative predictive factor in univariate analysis (Figure 2). Patients that had received the first whole brain irradiation prophylactically showed a significantly lower overall survival than those that had received the first WBRT therapeutically (p = 0.002). However, analyzing only the SCLC subgroup there was no significant difference in overall survival between those patients that received their first irradiation prophylactically and those that received it therapeutically.

**Figure 3:**
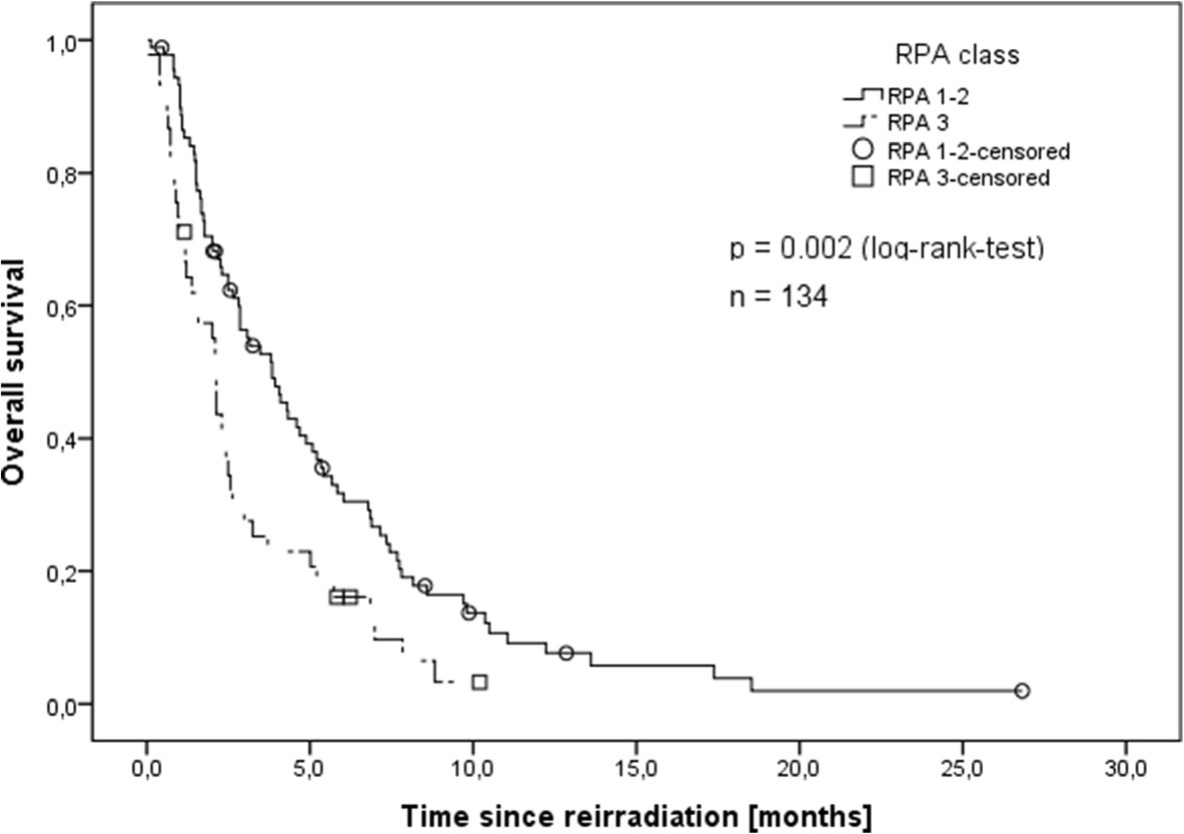
**Overall survival depending on RPA class (Kaplan-Meier’s estimation).** RPA class 3 with significantly worse prognosis (p = 0.002, log-rank-test).

Higher overall survival was found for patients whose primary tumors were controlled at the time of re-irradiation with median survival of 3.8 months while those without extracerebral control had a median of 2.4 months survival (p = 0.01). Another predictive factor was performance status at the time of re-irradiation. Patients with a Karnofsky performance status of ≥80 had a median survival of 4.9 months, compared to 3.1 months for those with a performance status of 70 and 2.1 months for those with a performance status of <70 (p < 0.001). These were both reflected in the RPA class with RPA class 3 being a negative predictive factor (Figure 3). Small cell lung cancer, primary tumor progression and low Karnofsky performance status were also identified as negative predictive factors in the multivariable Cox-regression analysis (Table 2) with more severe symptoms showing borderline significance. Male sex was a negative predictive factor in univariate, but not in multivariable analysis.

**Table 2:** Multivariable analysis

**Factor analyzed**	**Negative predictive factor**	**p-value**	**Hazard ratio (95% CI)**
Primary tumor	SCLC	0.019	0.59 (0.38 – 0.92)
Extracerebral control	No control of primary	<0.001	2.32 (1.47 – 3.66)
Karnofsky performance status	KPS <70	0.038	n/a**
Higher RPA class	RPA 3	n/a*	n/a**
Symptoms	Severe symptoms	0.070	n/a**
Interval between WBRT 1 and 2		0.557	1.12 (0.76 – 1.65)
Dose for re-irradiation		0.586	1.18 (0.65 – 2.16)
Male gender		0.087	1.45 (0.95 – 2.22)
Age ≥ 60		0.773	1.07 (0.69 – 1.65)

*Linearly dependent on Karnofsky performance status.

**Co-linearity between KPS, RPA and symptoms, therefore no individual hazard ratios.

Age, time between course 1 and 2 of the irradiation and dose of irradiation did not have any predictive value.

Only 28 patients (20.9%) could conduct the whole therapy as out-patients. 11 (8.2%) were at least partially treated as inpatients and 95 (70.9%) were completely treated as inpatients. Among these 95 patients, 45.3% were classified as RPA 3 and showed a Karnofsky-Index <70, compared to 3.6% in the group that conducted the whole therapy as out-patients.

Nevertheless, overall, Re-WBRT was tolerated satisfactorily. Main side effects were focal alopecia, fatigue, vertigo and erythema, few patients also showed more severe neurological symptoms like mnestic deficits, speech impairment or ataxia (Table 3). 14 patients (10.5%) had to discontinue treatment early with a slightly higher rate (12.6%) among those that were treated fully as in-patients.

**Table 3:** Rate of adverse symptoms after irradiation

**Symptom**	**n**	**%**
No adverse symptoms	44	33%
(Focal) Alopecia	29	22%
Fatigue	18	13%
Vertigo	16	12%
Erythema	15	11%
General weakness	14	10%
Mnestic deficits	13	10%
Difficulty swallowing, oral thrush	10	8%
Headache	10	8%
Nausea/vomiting	9	7%
Speech or sensory impairment	7	5%
Ataxia	3	2%

## Discussion

While several retrospective studies have analyzed the effects of re-irradiation in patients with brain metastases from solid tumors, the numbers of analyzed patients are low with no study consisting of more than 100 patients [9-16]. The largest collectives to date were analyzed by Wong et al. with 86 patients [14] and Sadikov et al. with 72 patients [16].

Over the years, radiation oncologists have become more generous when indicating a second course of WBRT, especially in patients where time to prior WBRT is longer and extracranial disease remains controlled. This trend is reflected also in our data, where a strong increase in patients/year treated with WBRT can be seen during the observation period.

Wong et al. and Sadikov et al. as well as most of the other studies come to the conclusion that repeat whole brain radiotherapy is efficient and safe enough to be accepted as a treatment that provides symptomatic relief to patients with a very limited prognosis. Our patient collective supports these results in showing that repeat whole brain radiotherapy can be an important therapeutic option with low rate of acute side effects for patients in adequate general condition. We have added our results to a summary first published by Sadikov et al. [16] and updated by Son et al. [9] to show an overview of previously published studies regarding re-irradiation of brain metastases (Table 4).

**Table 4:** Re-irradiation for cerebral metastases: overview of publications

	**Shehata et al. **[10]	**Kurup et al. **[11]	**Hazuka and Kinzie **[12]	**Cooper et al. **[13]	**Wong et al. **[14]	**Abdel-Wahab et al. **[15]	**Sadikov et al. **[16]	**Son et al. **[9]	**Present study**
n	35	56	44	52	86	15	72	17	134
Initial RT	1×10 Gy,	3×6 Gy	10×3 Gy	10×3 Gy	10×3 Gy	15×2 Gy	5×4 Gy	14×2.5 Gy	15×2 Gy
	10×3 Gy								
Re-RT	1×10 Gy	10×2 Gy	8×3.125 Gy	10×2.5 Gy	10×2 Gy	20×1.5 Gy twice daily (partial brain)	10×2.5 Gy	12×1.8 Gy	10×2 Gy
Interval	-	6.3 mo (mean)	7.8 mo (median)	> 4 mo	7.6 mo (median)	10 mo (median)	9.6 mo (median)	15 mo (median)	13.4 mo (median)
Response									
Improve	68%	75%	27%	42%	70%	60%	40%	80%	39%
Stable	25%	12.5%	41%	52%	29%	27%	33%	20% stable or no response	44%
None		12.5%	14%	6%			33%		17%
Toxicity	17% acute side effects	18% acute side effects 1 radiation necrosis	8 autopsies, 3 brain necroses	-	5 patients with radiographic abnormality		1 patient with severe side effects 3 unable to complete RT	71% with acute side effects	67% with acute side effects 10% unable to complete RT
Survival after re-irradiation	-	3.5 mo median	2 mo median	4 mo median	4 mo median	3.2 mo median	4.1 mo median	5.2 mo median	2.8 mo median

For the initial radiotherapy our median of 30 Gy in 15 fractions is in accordance with the doses and number of fractions used in most other studies. Shehata et al. [10], Kurup et al. [11] and Sadikov et al. [16] used a lower dose with a lower number of fractions with no obvious difference in outcome or toxicity. For the Re-WBRT in the majority of cases we applied 20 Gy in 10 fractions, a dose and number of fractions on the lower end of the spectrum of analyzed studies. Only Shehata et al. used a one-time irradiation of 10 Gy, while in all other cases 8–12 fractions were used. Abdel-Wahab et al. [15] analyzed the only patient collective where the Re-WBRT was applied as a partial brain therapy twice daily thereby duplicating the number of fractions.

With 13.4 months between first and second whole brain radiotherapy our interval was longer than that of most previous studies except for Son et al. with 15 months [9]. A reason for that could be the high number of patients with small cell lung cancer in our analysis that received the first whole brain radiotherapy prophylactically before they had developed brain metastases. Additionally, our internal guidelines recommend Re-WBRT only after a minimum of 6 months after primary WBRT, therefore rapidly progressive patients are not treated with Re-WBRT.

Response rates vary strongly between the different analyses. Hazuka and Kinzie [12] showed the lowest rate of improved symptoms with 27% while Son et. al. found improved symptoms in 80% of cases. These variances are probably due to the subjectivity of symptoms, especially if determined retrospectively based on patient charts and doctor’s notes. Our patients showed an improvement of symptoms in 39% of cases, stable symptoms in 44% and worsening symptoms in 17%, all numbers being fairly consistent with the responses found in other studies. A confounding factor in our as well as all other studies could have been the use of steroids that often improve the most severe symptoms before the radiotherapy has even started. However, due to the retrospective nature of the present analysis, detailed information on steroids, neuro-cognitive performance, quality of life and also imaging-defined progression-free survival are difficult to assess; also, KPS was only directly available for 50% of patients with the other being estimated due to performance as described in patient records; moreover, these endpoints are considered soft endpoints dependent on many clinical as well as personal factors, therefore, the focus of the present work was set on the hard endpoint survival.

While 10% of patients had to discontinue treatment before the planned dose was reached, treatment was generally tolerated well with minor side effects including as most common symptoms alopecia, fatigue, vertigo and erythema. Few patients showed more severe neurological symptoms like mnestic deficits, speech impairment and ataxia. The relatively low incidence of side effects might be due to the fact that a majority of patients received the first WBRT prophylactically with a common scheme of 15×2 Gy resulting in a lower equivalent dose than patients treated therapeutically with often 10×3 Gy. One limitation in assessing side effects is, that it is difficult to differentiate whether these symptoms were caused by the progressive metastases or the whole brain radiotherapy. Due to the progressive state of their disease and the resulting low performance status most patients had to be treated as in-patients.

Median survival after Re-WBRT was 2.8 months, the second lowest in all of the before mentioned studies. Median survival in the other studies was between 2 and 5.2 months. Only Kurup et al., Wong et al. and Son et al. also determined time to progression which was between 2.5 and 2.75 months. One reason why our survival is lower than that of most other studies could be the relatively high number of patients with SCLC that show a significantly worse outcome than patients with other primary tumors. This is likely also the reason why patients that had received the first whole brain irradiation prophylactically showed a significantly lower overall survival. A subgroup analysis of the patients with SLCS could not confirm the difference in overall survival between those patients that received their first irradiation prophylactically and those that received it therapeutically.

To evaluate the effects of different variables on overall survival, a stepwise backwards multivariable analysis was conducted. While the stepwise approach has certain limitations (Harrell in [17]), it still provides an important additional view, e.g., highlighting the fact that lower survival of male patients is likely due not to their sex, but to the higher prevalence of SCLC among this group.

As the RPA group is directly dependent on the KPI, no p-value could be obtained for this variable in the stepwise backwards analysis.

## Conclusions

While depending on histology and number of metastases WBRT is a standard of care in patients with newly diagnosed brain metastases as stated by the newest guidelines of the American Society for Radiation Oncology [18], treatment in the recurrent situation poses a therapeutic challenge. In the past, in general, no second course of WBRT was performed, due to the fear of treatment-related side effects. With our patient collective we could show that repeat whole brain radiotherapy can be an important therapeutic option with low rate of acute side effects for patients in adequate general condition.

Treatment should be weighed against supportive therapy with steroids alone, especially for patients with a low performance status that often have to be treated as inpatients.

For patients with a sufficient performance status, prospective, randomized studies with higher patient number will be needed to allow the better identification of patients that could benefit most from re-irradiation and to determine the ideal dose and fractionation. Therefore, the randomized ERASER-Trial comparing best supportive care, low-dose Re-WBRT with 20 Gy as well as Re-WBRT with 30 Gy is currently being prepared and will start recruitment soon.

## Competing interests

The authors declare that they have no competing interests.

## Authors’ contributions

HH participated in the design of the study and helped to conduct the statistical analyses. MS performed all analyses and drafted the manuscript. MB and JD participated in the design of the study and reviewed the results. SC conceived of the study, participated in its design and helped to draft the manuscript. All authors read and approved the final manuscript.
